# Supplementary material for: Polyketide synthase gene clusters in the ericoid mycorrhizal fungus Oidiodendron maius: genome-wide prediction, expression profiling, and functional characterization of knockout mutants
Source: Front Plant Sci. 2026 Sep 16;17:1924430. doi: 10.3389/fpls.2026.1924430 (PMC13623903; doi:10.3389/fpls.2026.1924430)
Supplement: Supplementary file 1 [file SupplementaryFile1.zip › Supplementary Materials/Supplementary Folder 1.zip!/Oidma1_AssemblyScaffolds_Repeatmasked/knownclusterblast/cluster44/jgi.p_Oidma1_135662_mibig_hits.html]

| MIBiG Protein | Description | MIBiG Cluster | MiBiG Product | % ID | % Coverage | BLAST Score | E-value |
| --- | --- | --- | --- | --- | --- | --- | --- |
| AAF81732.1 | putative\_FAD-dependent\_oxygenase\_EncM | BGC0000220 | Enterocin\_biosynthetic\_gene\_cluster | 30 | 97.4770642202 | 184 | 3.1e-46 |
| EAL92288.1 | FAD\_binding\_domain\_protein | BGC0000372 | Hexadehydro-astechrome\_(HAS)\_biosynthetic\_gene\_cluster | 30 | 101.146788991 | 166 | 6.7e-41 |
| ABP55175.1 | FAD\_linked\_oxidase\_domain\_protein | BGC0000150 | Sporolide\_biosynthetic\_gene\_cluster | 25 | 91.0550458716 | 130 | 5.3e-30 |
| EHA28234.1 | FAD/FMN-containing\_dehydrogenase | BGC0001143 | Azanigerone\_biosynthetic\_gene\_cluster | 27 | 66.0550458716 | 119 | 7.2e-27 |
| ACB37752.1 | dehydrogenase | BGC0000162 | Tetrocarcin\_A\_biosynthetic\_gene\_cluster | 35 | 48.8532110092 | 111 | 2e-24 |
| ABI22129.1 | oxidoreductase | BGC0000422 | Saframycin\_A\_biosynthetic\_gene\_cluster | 39 | 36.9266055046 | 108 | 2.2e-23 |
| AAM33667.1 | putative\_FAD-binding\_oxidoreductase\_GrhO1 | BGC0000230 | Griseorhodin\_biosynthetic\_gene\_cluster | 37 | 37.6146788991 | 105 | 1.8e-22 |
| ADY16695.1 | TqaG | BGC0001142 | Tryptoquialanine\_biosynthetic\_gene\_cluster | 31 | 44.7247706422 | 104 | 4.1e-22 |
| ACB46474.1 | FAD-dependent\_oxidoreductase | BGC0000082 | Kijanimicin\_biosynthetic\_gene\_cluster | 35 | 47.0183486239 | 99 | 1e-20 |
| BAD83683.1 | FAD/FMN-dependent\_oxygenase/oxidase | BGC0000012 | Alternapyrone\_biosynthetic\_gene\_cluster | 36 | 36.4678899083 | 96 | 6.5e-20 |
| ABI22120.1 | oxidoreductase | BGC0000422 | Saframycin\_A\_biosynthetic\_gene\_cluster | 33 | 44.7247706422 | 92 | 1.6e-18 |
| AHG26149.1 | FAD-linked\_oxidoreductase\_YvdP-like\_protein | BGC0000812 | Huperzine\_A\_biosynthetic\_gene\_cluster | 30 | 43.119266055 | 91 | 3.6e-18 |
| KGO40470.1 | FAD\_linked\_oxidase,\_N-terminal | BGC0001205 | Communesin\_biosynthetic\_gene\_cluster | 33 | 39.9082568807 | 84 | 3.4e-16 |
| AAM78001.1 | oxidase | BGC0000112 | Neocarzinostatin\_biosynthetic\_gene\_cluster | 32 | 45.1834862385 | 80 | 4.8e-15 |
